# The Vulnerability and Resilience of Drinking Water Systems to Extreme Weather Events and Future Climate Change

**DOI:** 10.1007/s40572-026-00524-y

**Published:** 2026-02-05

**Authors:** Guy Howard, Lindsay Beevers, Katrina Charles, Anisha Nijhawan

**Affiliations:** 1 School of Civil, Aerospace and Design Engineering and Cabot Institute for the Environment, University of Bristol, Queens Building, University Walk, Bristol, BS81TR UK; 2 School of Engineering, University of Edinburgh, Edinburgh, UK; 3 School of Geography and the Environment, University of Oxford, Oxford, UK

**Keywords:** Drinking water, Climate resilience, Water safety, Water management, Risk management, WSPs

## Abstract

**Purpose of Review:**

We reviewed the evidence on climate resilience of the drinking water sector, focusing on: How are climate hazards affecting drinking water supplies changing? How is resilience measured? What interventions are being used to build resilience?

**Recent Findings:**

The frequency and intensity of flooding and drought are increasing, water quality is deteriorating, and wildfire and sea-level rise pose increasing threats. Frameworks to measure resilience are emerging, but none is applied universally. a wide range of actions are required to build resilience but there is limited evidence of uptake and performance. Non-utility water supplies are at particular risk but investment in resilience is limited.

**Summary:**

Climate change poses a major threat to drinking water supplies, but current actions to improve resilience are insufficient. More evaluations of the performance of resilience measures are needed. Floods and drought remain the most studied threats, but risks from wildfire, water quality and sea-level need more attention and research. More work is needed to consolidate how resilience is measured. A summary of the detailed findings in provided in Table 1 at the end of the review.

## Introduction

The provision of safe drinking water in sufficient quantities is central to protecting public health and is the focus of Sustainable Development Goal 6. Global statistics indicate that around 75% of the global population has access to what is defined as ‘safely managed water’ [1]. However, the data on which this assessment is made does not include detailed information on water quality and it is likely that far fewer people actually enjoy safe drinking water [2]. This contributes substantially to the ongoing high burden of disease attributed to inadequate water, sanitation and hygiene [3, 4].

**Fig. 1:**
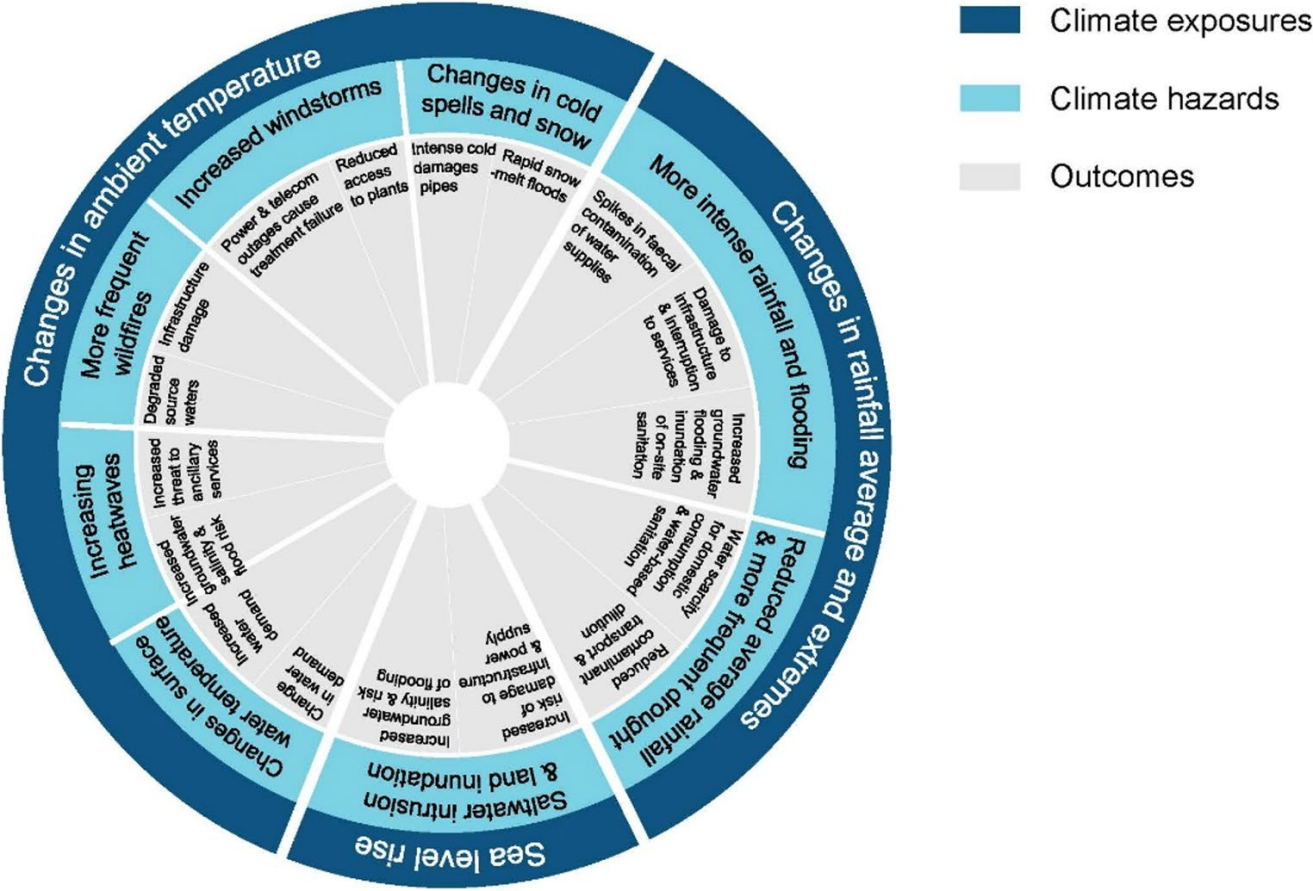
The climate wheel of impacts

Water supplies are at risk from climate change, with a range of climate hazards and their effects posing challenges to maintaining safe drinking water [5]. Figure 1 below illustrates the range of climate hazards and their effects on water supplies.

Even in well-regulated and managed systems, climate hazards can lead to damage to infrastructure and temporary loss of supply or lead to contamination events and outbreaks of disease [6]. Poorly managed and unregulated systems are frequently affected by climate-related hazards and events, leading to increases in endemic disease rates and patterns as well as outbreaks of water-related disease [7, 8].

As the climate changes because of global heating, the risk of diarrhoeal disease caused by poor water supply is likely to increase given the well-established associations between climate hazards and outbreaks of disease [9–11]. However, there are increasing risks from exposure to toxic chemicals in flood waters [12]. A recent systematic review suggested that waterborne diarrhoeal disease is likely to increase with increases in mean temperature and rainfall, although this was primarily found for use of unimproved facilities and did not capture changing risks in improved water supplies [13]. A further review showed that changes in climate will be likely to increase the persistence of key waterborne pathogens thus increasing the challenges of ensuring water safety [14]. Increasing temperatures will create conditions that favour proliferation of opportunistic pathogens, commonly found in biofilms within pipes in the future [15]. In addition to infectious disease, rising temperatures will increase threats of dehydration [16]. This is already noted as contributing to increased prevalence of chronic kidney disease [17].

In this paper we review the current state of knowledge about the resilience of drinking water supplies across the world. We review how key climate hazards that affect water supplies are changing, before reviewing current approaches to measure resilience and the types of action being taken to build resilience. Finally, we discuss what this evidence tells us about current resilience and what more needs to be done.

## Climate Impacts on Water Supplies

Droughts and water scarcity are the most common and generally considered the greatest threat for water supplies. Changes in rainfall patterns and temperature increases are shifting the occurrence of droughts. Meteorological and hydrological droughts are typically slow onset and long duration events, although the impact of flash droughts on streamflow being increasingly recognized [18]. The scale of drought impacts is considerable and is increasing, affecting large areas and populations, requiring proportionate resilience approaches. An increase in agricultural and ecological droughts has been observed in many regions across Africa and Europe, with low agreement on the change in most of the rest of the world [5]. Recent data shows the areas in drought have expanded by 74%, affecting 30% of global land area in 2022, with water demand from the heating atmosphere a significant driver [19].

The impacts of reductions in rainfall on hydrology and water systems are closely linked to how land and water systems are managed. Actions in catchments and in the engineered water systems can greatly influence exposure. Drought risks are enhanced where catchments have been allowed to degrade. Across the catchment, land use impacts the retention and storage of water that maintains access during periods of low rainfall, as well as evapotranspiration and abstraction rates that can accelerate the impact of droughts. The impacts of drought can compound financial sustainability issues where reductions in water use result in reduced water service revenue collection, such as occurred in Cape Town [20]. This may undermine long-term sustainability and viability of supply.

Fewer studies concentrate on flood risk, despite water supply infrastructure often being located on river floodplains or in coastal areas [21, 22]. Global flood exposure has increased between 1975 and 2015, with much of this increase occurring in urbanised areas, where settlements have grown within flood risk zones [23]. Much of the existing exposure is due to streams and small rivers (catchments up to 1000km^2)^ emphasising their importance for managing resilience alongside the larger global rivers [22, 23]. This was highlighted in the severe European floods in the summer of 2021 when drinking water supply and sanitation systems were severely damaged; with homes having their supply fully reconnected only within a month (Belgium) and up to 2 months later (Germany). Impacts to water supply was reportedly from both the failure of the infrastructure (during the flood), as well as pollution of the supply by the floodwaters. In the case of Belgium the re-construction of the infrastructure took 6 months to deliver [22]. Similar impacts were reported in western North Carolina, USA, as a consequence of Hurricane Helene which hit on 27 September 2024. Helene damaged or destroyed 163 sewage and water treatment systems in multiple communities which contributed to over 10% of the overall direct damage bill [24]. Reconstruction of these facilities is still ongoing.

As the climate warms, air can hold more moisture, thus climate change will increase the frequency and intensity of flood hazards globally; with more intense rainfall occurring in short periods of time. Estimates suggest that with high end warming scenarios global flood risk from all sources could double by the end of the century [25]. It is not only climate change which can drive changes in increasing flood exposure, the way we manage the land within catchments also has a significant effect on extreme river flows. Land use policies ranging from agricultural policy, land drainage, through to renewable energy provision (e.g. new wind farms, or hydropower installations) can change river flows [26]. If land use is carefully managed it can increase flood risk downstream. However perhaps the clearest example of managing exposure is the location of critical infrastructure, which is often found in known floodways.

Multiple reviews have synthesized evidence on links between climate stresses and source water quality and drinking water supplies [27–29]. Shallow groundwater sources often fail during prolonged drought because of localised aquifer depletion, affecting user behaviour [30]. Heavy rainfall events, which may become more common in many parts of the world, may increase groundwater contamination, especially in communities with open defecation and poor sanitation coverage. Wildfires may affect catchment hydrology and increase dissolved organic carbon and precursors for disinfectant by-products [27]. Erratic rainfall patterns may also affect geogenic contaminants, although this evidence is not robust, while tropical storms and sea-level rise make coastal groundwater more saline [29, 31].

### Cascading Risk and Compound Hazards

Drinking water supplies can be vulnerable to other ‘upstream’ risks and in particular to ancillary services such as power, telecoms and roads [7]. Some forms of power generation rely on adequate water for cooling and heatwaves in Europe have demonstrated how water supply shortages affect generation, which may in turn reduce power available to run drinking water supplies [32]. The effect of major storms on water supplies may result from loss of these services rather than direct effects, meaning extended loss of supply, which may particularly affect rural supplies [33]. Furthermore, hazards that disrupt water supplies have ‘downstream’ effects, most importantly in terms of risks of preventable disease, but may also impact the environment from waste produced during water treatment [34].

In addition to individual hazards, there are concerns about what are termed as compound hazards. Compound hazards are where two or hazards related to climate change interact and influence the overall effect [35]. This may happen for instance when extreme rainfall occurs after a period of drought that raises the risk and impact of flooding, or when surface flooding occurs at the same time as fluvial or coastal floods [22, 36]. Such events increase the risk of damage to water systems or lead to excess levels of contamination in source waters such that treatment processes are compromised. Other compound hazards of relevance to water supply are excess rainfall causing landslides in dammed catchments that lead to dam breaches and loss of supply and damage to downstream infrastructure. Both heavy rainfall and increasing temperature individually and combination increase risks of harmful algal blooms which compromise water treatment processes and which may contain toxins [29].

## Drivers of Water Supply Vulnerability

The drivers of vulnerability of drinking water systems start from the design and management of systems and catchments. Systems that are well-designed, properly managed in catchments that are protected and well-managed are inherently less vulnerable than those that are not. Ensuring that there are proactive programs of leak detection and rapid, as well as schedule system maintenance are particularly critical in reducing the impact of drought and preventing contamination. Equally, catchments should be properly managed to reduce the risks of flash flooding and contamination of water sources.

The changes in climate already being seen will require better design of systems to ensure greater buffers in areas of drought and protection from floods and cascading hazards. For drought management, there will need to be investment in re-use of treated wastewater, development of new large-scale storage, inter-basin transfers and use of multiple sources [7, 37]. Coastal areas present particular challenges as source waters become more saline as sea levels rise leading to consideration of moving intakes inland, developing desalination of actions in catchment management and managing risks of corrosion or other damage to piped systems [38, 39].

### Regulation and User Behaviour

Regulation and governance will increase vulnerability through two key mechanisms: over-stretching the resource and restricting adaptability. Over-stretching relates to the way regulations can reduce the availability of water of appropriate quality through over allocation and not protecting against over-abstraction or pollution. In Dhaka, inadequately regulated discharges of wastewater lead to high concentrations of industrial pollution in the dry season which have required construction of alternative water supply facilities to reliably access water that can be readily treated for drinking [40]. The combination of poor wastewater management leading to increasing nutrients in water and increasing changes in temperature and precipitation are increasing threats of harmful algal blooms, with consequent challenges for water treatment and elevated health risks [29, 41]. Over-abstraction is a common issue which compounds drought impacts. In Ethiopia, this has been exacerbated by poorly regulated self-supply for industrial users reducing groundwater availability in shallower water systems [42]. Adaptations to reduce abstraction have often underperformed due to the complexity of incentives and regulation needed [43].

Regulations further increase vulnerability when they reduce the potential to adapt to extreme events. Prioritization of drinking water is common in regulations, but there can be limited means to ensure changes in other uses to protect drinking water access, depending on the local governance. As the quantity and quality of water changes, abstraction volumes will need to vary. Regulations can limit abstractions by user groups or type [44]. Market approaches to managing competing water uses with agriculture are one approach to move from static allocations to allow relatively rapid shifts in water abstractions [45]. A lack of data for decision making will further increase vulnerability, both data for adapting to long term trends and short-term data to design responses in a crisis. Poor data on the rural water sector, often not prioritized in regulatory implementation, creates a significant gap in understanding and addressing vulnerabilities.

Users of drinking water, both at the household and commercial scale, further enhance vulnerabilities. Attitudes to outside water use during droughts vary by culture: in the UK, ‘hosepipe bans’ limiting outdoor water use are perceived as a failure of the water utility rather than an opportunity to shift perceptions on the value of water. Evidence from Australia shows increased household water conservation but potentially reduced by perceptions of resilience from multiple sources [46]. Increasing use of water-based sanitation, washing machines and dishwashers locks households into a reliance on piped water, increasing vulnerability when piped systems can’t operate. People with high trust in their water supplies may also lack awareness of how to manage hazards when they arise, requiring strong public communication.

## How Well Do We Measure Resilience?

In the last decade, multiple frameworks have been developed to improve our understanding of climate impacts and the actions that can mitigate them. Building on definitions of resilience, including the IPCC, Sanitation and Water for All define resilient water and sanitation services as those that: ‘*anticipate*,* respond to*,* cope with*,* recover from*,* adapt to or transform based on climate-related events*,* trends and disturbances*,* all while striving to achieve and maintain universal and equitable access to safely managed services*,* even in the face of an unstable and uncertain climate*,* where possible and appropriate*,* minimising emissions*,* and paying special attention to the most exposed vulnerable groups’* [47].

In high income countries, several examples exist of methods to assess resilience used for utility supplies and to a more limited extent small systems [48–50]. In some high-income countries, regulators demand regular reporting on actions taken by water utilities to build resilience but rarely cover non-utility water systems.

Other frameworks have been developed focused on low- and middle-income countries. GWP and UNICEF developed an updated Strategic Framework for WASH Climate Resilient Development which recommends climate risk assessments to improve resilience and has been applied in South Africa [51, 52]. The *How Tough is WASH* framework uses a systems approach and with indicators measuring resilience along six domains [53]. These indicators, similar to WASH building blocks cover multiple aspects of service delivery and have been used to identify factors linked to the response of rural water supplies to climate extremes [29, 54–56]. Integrating climate resilience into WASH system building block tools has also been proposed by ISF-UTS and SNV, WaterAid and Water for Women. Other frameworks focus on a specific aspect of services [57–59]. Climate Resilient Water Safety Plans [60] incorporate climate risks into traditional Water Safety Plans, focusing on technical and infrastructural measures to mitigate these risks.

Water and sanitation also feature in frameworks designed to assess resilience at the city level. Key examples are the City Resilience Framework which assesses resilience of strategic urban services: water supply, wastewater and storm water and those closely related with the water services such waste management, electrical energy supply and mobility. Focus on climate change and the water cycle is made through multiple resilience dimensions including governance, environment and infrastructure; and the City Water Resilience Framework [61, 62] that can also be used to assess the resilience of urban water systems in a city, with a focus on governance functions of the urban water system.

The increasing interest in measuring resilience and the different ways being adopted to do so has led to efforts to try to harmonise monitoring. There is an ongoing process to support the identification and integration of resilience measures into global monitoring of SDG 6. Similarly, the UNECE-WHO Protocol on Water and Health noted the importance of a single metric for resilience assessment across the pan-European region [63]. Developing a common standard methodology would be an important step forward in both improving our understanding of progress in building resilience and in facilitating knowledge exchange and capacity-building. More work is also needed to incorporate health measures into resilience monitoring frameworks, as these remain largely absent.

## Building Resilience

The previous sections have covered the nature of the hazards that affect water supplies, the factors that increase vulnerability or may increase resilience, and current attempts to measure resilience. In this final part of our review, we provide a brief overview of the evidence of actions taken to improve resilience.

There are a wide range of specific interventions that can be used to build resilience which may be found in HICs and LMICs [29, 49, 55]. These cover aspects around water source and resource management, actions to better safeguard distribution systems and water treatment plants and policy interventions [36, 64]. Some of the adaptation actions fall under the direct responsibility of service providers, whereas others, notably new reservoir construction or major inter-basin transfers may fall under national critical infrastructure investments. Equally important may be actions to reduce demand among users, particularly where drought is a growing risk [44].

While specific actions have been identified in individual water supplies, there is more limited evidence of widespread integration of climate adaptation measures in water utility management. A study of water utilities in the US, for instance, demonstrated high awareness of climate change among utility managers but limited integration of comprehensive climate adaptation plans [65]. This reflects earlier trends for the limited uptake of weather forecasts, with widespread challenges in uptake of climate information in water management [66, 67]. In the UK, assessments in adaptation have identified that insufficient progress is being made to increase resilience in water supplies [68].

A global review of adaptation to drought in utilities noted that while the vast majority of utilities would benefit from adaptations, only 5–20% of adaptations would have a benefit-cost ratio of greater than one [69]. This illustrates a common problem that the combined uncertainties of climate threats and those associated with likely impact of interventions, means that economic analysis of adaptation options often pushes decisions to some point in the future [70]. This can then make it difficult to attract the finance required to make necessary investments [68].

This can be addressed through more scenario-based planning, for instance the CREAT tool developed by the U.S. Environmental Protection Agency assists utilities in understanding and addressing climate change risks and helps identify decision-points [71]. World Bank resources for technical assistance to build resilience in utilities include a broad approach to plan for and evaluate the impact of climate stressors to water and sanitation utilities and a design brief that focuses on incorporating resilience to floods, droughts and high winds into the engineering design of water and sanitation infrastructure [72].

The integration of climate resilience into water safety plans has emerged as one strategy that is widely promoted with some limited evidence on uptake [73]. Examples from high-income countries such as Italy and Scotland show how climate hazards can be integrated into the detail of a water safety plan [74, 75]. There are fewer examples from LMICs, partly reflecting the slower uptake of WSPs in general, but examples exist of attempts to integrate climate change into WSPs that do exist [76, 77]. Integrating climate into WSPs would seem to be a useful option moving forward, but this is likely to pose new challenges in auditing effectiveness.

Catchment based solutions can enhance the resilience of drinking water supplies by buffering the impacts of climate change on hydrological regimes when planned in a holistic manner. Catchment solutions can be a mixture of hard engineering solutions (for example reservoirs for drinking water supply which store water during high flow periods and release it during droughts) and nature-based solutions [78]. Nature based solutions can provide complementary and sustainable benefits for climate resilience which act at a local level buffering pollution (e.g. through wetlands or buffer strips), storing water during precipitation events (e.g. catchment reforestation or afforestation, re-meandering of rivers, peatland restoration), and addressing water scarcity (e.g. micro-reservoirs, riparian bunds to encourage groundwater recharge) whilst also providing solutions which can regulate temperatures and provide cooling effects. Challenges exist however in the uptake of catchment and nature-based solutions which arises from a lack of consistent and specific evidence on their impact to the hydrological regime, alongside further research into the process understanding of the interventions [79].

Much of the above reflects actions taken by utility supplies, operated by professional skilled staff. Where supplies are managed by professionals, there are more opportunities to build climate resilience and alignment to national approaches. There is very little evidence of action to build resilience of non-utility supplies that are typically managed by communities or in some cases individual households. This is a particular problem in LMICs given the large number of such supplies [7, 55]. However, such supplies are also found in HICs and there is a similar trend of little evidence of action [80].

## Summary of the Review Findings

Table 1 below summarizes the key findings from this review.

**Table 1:** Summary of the review findings

Climate threats to water supplies	
Drought, floods, increasing temperature, wildfire, sea-level rise, glacier recession, cascading and compound risks	Increasing threats from pathogens, including opportunistic pathogens in piped systems, loss of supply and hygiene-related disease, exposure to increase chemical contamination, promotion of harmful algal blooms and associated health risks
Drivers of vulnerability	
Poorly managed catchments, weak operation and maintenance, weak regulation, user behaviours, small or private water supplies.	Increasing threats to health from supply disruption and contamination of supplied drinking water. Community-managed supplies in LMICs and private supplies in HICs of particular concern.
Measuring resilience	
A range of metrics and frameworks developed, with greater and lesser complexity; need to consider a range of environmental, social, and engineering factors; lack of consensus on indicators.	Substantial attention now being paid to assessing resilience, but resilience is difficult to measure, and robust proxies are needed. There is a need to develop common methodologies to support lesson-learning and benchmarking. Need to get more attention to health measures within monitoring.
Climate actions	
Climate-resilient water safety plans; improved catchment management; redundancy (multiple water sources), drought resilience plans; regulations; nature-based solutions.	Evidence indicates insufficient progress globally. Specific interventions often lack robust evaluations and limited data and so it is hard to assess their impact. Cost-effectiveness of resilience actions is often difficult to assess and these result in deferred decisions. Small/private water supplies have least action.

## Conclusion

Drinking water supplies are already at substantial risk from climate change and these threats are increasing. There are a range of climate hazards that may affect water supplies, with drought and floods being the most commonly considered and studied. Water quality deterioration is also important, if less comprehensively studied, while coastal areas are at additional risk from sea level rise. Measurement of resilience remains fragmented and there is no standard set of measures that is widely adopted. This makes systematic assessment of progress difficult. While the recognition of the growing threat is widespread, there is little evidence of sustained, sector-wide actions being taken, despite many interventions being well understood and offering wider benefits. Adaptations will inevitably need to reflect local context, but the overall picture is concerning and indicates a sector that is failing to take the concerted action required.

## Key References

- Murgatroyd A, Gavin H, Becher O, Coxon G, Hunt D, Fallon E, et al. Strategic analysis of the drought resilience of water supply systems. Phil. Trans. R. Soc. A, 2022, 380: 20210292. 10.1098/rsta.2021.0292.
  - ○ Critical analysis of drought resilience and selection of key measures to ensure resilience.
- Colston JM, Zaitchik BF, Badr HS, Burnett E, Ali SA, Rayamajh A, et al. Associations between eight earth observation-derived climate variables and enteropathogen infection: an independent participant data meta-analysis of surveillance studies with broad spectrum nucleic acid diagnostics. Geohealth 6: e2021GH000452. doi: 10.1029/2021GH000452.
  - ○ Study that demonstrates how the persistence of different pathogens is influenced by climate variables and therefore how risks of dairrhoeal disease may change as the climate warms.
- Lyle ZJ, VanBriesen JM, Samaras C. Drinking Water Utility-Level Understanding of Climate Change Effects to System Reliability, ACS ES&T Water, 2023, 3 (8), 2395-2406. 10.1021/acsestwater.3c00091.
  - ○ Paper critically examines current thinking and action on resilience in utilities across the USA.
- Rickert B, van den Berg H, Bekure K, Girma S, de Roda Husman AM. Including aspects of climate change into water safety planning: Literature review of global experience and case studies from Ethiopian urban supplies. Int J Hyg Env Health, 2019, 222(5): 744-755. 10.1016/j.ijheh.2019.05.007.
  - ○ Paper that provides a review of progress with integrating climate resilience into water safety plans at a global level, with examples of how this is done in practice.
- Khan SJ, Deere D, Leusch FDL, Humpage A, Jenkins M, Cunliffe D. Extreme weather events: Should drinking water quality management systems adapt to changing risk profiles? Wat Res, 2015, 85: 124-136. 10.1016/j.watres.2015.08.018.
  - ○ Review that demonstrates the challenges to managing safe water supply in extreme weather and needs for future research.
- Nijhawan A, Howard G. Associations between climate variables and water quality in low- and middle-income countries: A scoping review. Wat Res, 2022, 210: 117996.
  - ○ Scoping review demonstrating how climate variables and climate change affect water quality.

## Data Availability

No datasets were generated or analysed during the current study.
